# A Framework for Developing Evaluation Tools Used in Washington State’s *Healthy Communities* Projects

**Published:** 2006-03-15

**Authors:** Donna B Johnson, Lynne T Smith, Erica Lamson, Marilyn Sitaker

**Affiliations:** Center for Public Health Nutrition, University of Washington; Center for Public Health Nutrition, University of Washington, Seattle, Wash; Center for Public Health Nutrition, University of Washington, Seattle, Wash; Cardiovascular, Diabetes, Nutrition & Physical Activity Section, Washington State Department of Health, Olympia, Wash

## Abstract

Washington State's *Healthy Communities *pilot projects were developed to test approaches and recommendations of the Washington State Nutrition and Physical Activity Plan and to provide a statewide model for implementation. The *Healthy Communities* program included plans for ongoing process evaluation to ensure implementation. Two years into the first project, however, the evaluation team recognized that data for evaluation were inadequate to explain the experiences of the pilot community partnership. The team sought a framework through which to better understand how the community partnership functioned, including what worked well and how guidance and technical assistance could best be provided. The evaluation team identified the community health governance model of Lasker and Weiss through a literature search and applied this model to existing *Healthy Communities *project evaluation data. The team also designed a new survey tool based on the model and used it in the second pilot community. The new tool provides feedback to community partners to help guide project implementation and tests the applicability of a theoretical model to public health practice.

## Introduction

Public health practitioners are increasingly being asked to partner with people in nonhealth sectors of the community to develop policies and build environments that will support health-promoting nutrition and physical activity behaviors (1-7). For example, *Preventing Childhood Obesity* (2), a recent report by the Institute of Medicine, recommends that state and local governments work with communities to expand and support opportunities for physical activity and access to healthy foods. Programs for successful obesity prevention and reduction need to be multilevel, community based, and sustainable (2,8). Populationwide changes in behaviors require interventions that address policies that affect nutrition and physical activity environments (5,9).

Critical factors for successful policy and environmental change include collaboration, support from community decision makers, and data that favor the intervention, in addition to funding, other resources, and skilled staff (1). Successful collaborations and community support require durable planning structures and *social capital* or *social readiness* (10,11). The elements of social capital — social relationships, social networks, social norms and values, and trust — reflect dynamic processes and interactions (12). Meaningful assessment of social capital accounts for its dynamic nature and is more complex than simply quantifying how many people are involved and which agencies are represented.

Community partnerships are part of social capital. Understanding characteristics of a community partnership provides insight into how an active partnership is functioning and how this functioning affects project implementation. Public health practitioners typically evaluate community partnership projects by identifying actions and outputs that measure whether a project is meeting its objectives and goals. Equally important but perhaps more difficult to evaluate is the nature of the partnership itself — the ways that members come together and interact and how the work of the project is accomplished. Public health practitioners need to understand community partnerships as social capital to provide guidance for setting goals and objectives and to give appropriate technical assistance. In this article, we describe the evolution of methods and tools that were used to identify and understand the characteristics, structures, and processes of a community partnership for health improvement in Washington State.

## Washington's *Healthy Communities *Pilot Projects

The Washington State Department of Health (DOH) has lead efforts to prevent obesity and overweight on both the state and community policy levels. Community efforts have been organized as *Healthy Communities* projects. With funding from the Centers for Disease Control and Prevention (CDC), DOH recruited the city of Moses Lake in 2002 as the first pilot community to test implementation of the Washington State Nutrition and Physical Activity Plan (13). Table 1 provides a timeline of the *Healthy Communities *projects.

Initial planning for the Moses Lake pilot project was conducted by an advisory committee composed of leaders and representatives of civic organizations, city and county agencies, businesses, and interest groups as well as community residents. Members of the advisory committee identified three initial strategies from the state plan and created and formalized an action plan for their implementation. In the next phase, activity shifted from the advisory committee to the three project teams: 1) trails and paths, 2) breastfeeding, and 3) community garden. Many members of the advisory committee served on a project team and recruited new members. During this phase, the advisory committee as a whole met less frequently. The leadership group (planning team), including the project coordinator, advisory committee, three team leaders, and DOH staff, continued to meet to coordinate the subprojects and monitor overall progress.

The evaluation team for *Healthy Communities* included staff from the University of Washington (UW) and DOH. Because our initial tools did not adequately explain the Moses Lake pilot project experiences, we searched for a theoretical framework that would include constructs that describe factors we identified as barriers to or enhancers of the success of *Healthy Communities Moses Lake*. We conducted a literature review of available frameworks and identified the community health governance (CHG) model developed by Lasker and Weiss (14) as a best fit for existing data from Moses Lake. The CHG model served as the basis for an evaluation tool that was applied to the second *Healthy Communities* pilot project, the city of Mount Vernon, from its beginning.

Mount Vernon was selected as the second pilot city in 2003. The Mount Vernon project's assessment, planning, and project team organizational phases were similar to those of Moses Lake, although the projects and composition of committee partnerships were different. DOH and the pilot project communities also had distinct objectives and deadlines according to their interests.

## Developing the Evaluation Tool

Data collected in the first 2 years of the *Healthy Communities* project were used for qualitative and quantitative process evaluation. These data were collected through two telephone interview surveys of advisory committee members and a survey of project team members from each project: the trails planning team, the Breastfeeding Coalition, and the community garden team. Supplementary data included observations during meetings and events, meeting evaluations and minutes, and debriefing of project staff (Table 2). The initial evaluation plan for *Healthy Communities Moses Lake *was developed in partnership with action-oriented stakeholders. It examined some aspects of collaborative processes but was not based on an explicit overarching theoretical model.

The UW evaluation staff invited *Healthy Communities* participants to respond to the surveys. The surveys were designed to be as brief as possible while providing sufficient detail on partnership functioning, issues of interest to advisory committee members, processes of program planning and implementation, and use of technical assistance to inform the public health practitioners. Survey results were compiled and reported to DOH staff, who shared the information with the respective community leadership group that in turn reported to its advisory committee.

### First survey, *Healthy Communities Moses Lake*

The purposes of the first Moses Lake project survey, conducted in December 2002, were to 1) provide feedback to the project leadership team so that the community development process could be improved and 2) gather ongoing needs assessment data from new stakeholders in Moses Lake. The survey consisted of 14 scaled questions that asked members of the advisory committee to evaluate the committee structure and function, leadership facilitation, technical assistance that was provided, their commitment and personal values, and their understanding of the project's purpose and goals. Open-ended questions asked about partnership values as well as barriers to and motivators for participation; these questions also provided an opportunity to identify other people who might want to be involved in the project.

A survey of Moses Lake advisory committee members was conducted again in January 2004. This survey was similar to the December 2002 survey except that it included new questions to identify partnerships that had been formed among committee members and new questions to measure the degree of integration of *Healthy Communities *work across agencies and programs.

### Second survey, *Healthy Communities Moses Lake*

In the second year, the focus of *Healthy Communities Moses Lake* shifted from the advisory committee to the three project teams. During this time, the membership fluctuated, and the project evaluation team asked additional questions to better understand the reasons for the fluctuation. In June 2004, a new telephone survey was conducted that included most of the questions from the December 2002 survey as well as additional open-ended questions that asked about members' motivation, perceived support, priorities, barriers, project impact, costs and benefits, and understanding of project purpose. The full survey was given to members of the three project teams. In addition, three advisory committee members who had responded to the survey in January 2004 were selected to answer only the new set of open-ended questions.

The survey results provided feedback about how the project was progressing and how the partnership was functioning, and the new set of open-ended questions added qualitative information about members' experiences. However, the data did not systematically integrate the reported experiences with other reports (Table 2) of how the partnership functioned.

### Use of CHG model to revise survey tool

At this time, the evaluation team began to use the CHG model (14) as a way to organize observations and systematically examine partnership functioning in a *Healthy Communities *project. In the CHG model, *critical characteristics* take into account who participates in a project and how they participate in the collaborative process. The *proximal outcomes* of the partnership process are the empowerment of individuals and groups to come together in partnership, create and enhance social ties, and work to resolve community health problems. The roles of *leadership and management* are crucial to ensuring that the critical characteristics of the collaborative partnership process can occur. Leadership and management roles include promoting active participation that is broad and representative of the community, facilitating the group processes, promoting incremental growth and development of the partnership, and providing training and technical assistance as needed. The CHG model elaborates on examples of these elements of the collaborative partnership process.

Eleven of the scaled questions in the two Moses Lake surveys touched on components of the CHG model, including individual empowerment, bridging social ties, synergy, critical characteristics of who was involved and how they were involved, the scope of the process, and leadership and management parameters, including promoting participation and facilitation. However, the survey questions did not provide adequate data to apply an integrated overview of the CHG model, and the survey was substantially revised to include additional CHG constructs (Table 3).

### Revised survey, *Healthy Communities Mount Vernon*

The second *Healthy Communities* project began in Mount Vernon in January 2004, and the revised survey tool was administered to members of the Mount Vernon advisory committee in September. The survey was administered at the end of the committee's planning phase before it launched its three projects. The purpose of the new survey was to be able to understand how leadership, management, and process and community dynamics contributed to the long-term success or failure of initiatives to improve community health. In the revised survey, the focus shifted from individuals' experiences and perceptions to their views of the partnership itself and how the partnership functioned.

The evaluation team used published studies of community partnership functioning and related survey tools to create the new survey (Table 4). The revised survey included 45 scaled questions and 3 open-ended questions. Although several of the scaled questions were carried over from the two previous versions of the survey, all of the scaled questions were included to address elements of the CHG model (Table 3). The new questions differed from several previously published tools (Table 4) by using language particular to the given community project rather than more general terms. For example, questions referred to the name of the committee or the names of agencies that provided technical assistance. In addition, the new survey included a few open-ended questions to collect more subjective input and supplement the scaled questions. Open-ended questions asked for comments about community representation on the project, barriers to participation, and any other issues that members wanted to address. To obtain feedback on membership turnover, the evaluation team added additional open-ended questions about barriers to participation for members who had attended only one meeting and who no longer appeared to be active in the project. 

The survey was easy to administer and score, and conducting the survey by telephone permitted evaluation staff to clarify questions and take notes of comments and feedback. Most of the UW staff who administered the survey reported that the questions seemed to be readily understood. The survey took 20 to 25 minutes to complete.

For all of the surveys, triangulation was an integral part of data analysis. Results of the surveys were compared with objective observations of the project, including community involvement, attendance and participation at meetings, and progress toward project objectives and goals (Table 2). Overall, the results of the survey in Mount Vernon indicated strong positive functioning in multiple levels of the partnership processes. These results were consistent with observations by various partners and resonated with the leadership group.

Results were used for further planning. For instance, responses to questions about *leadership and management for* *broad and active participation* indicated that Hispanic residents were not adequately represented on the advisory committee. The advisory committee had recognized from the outset the need to include diverse groups in the community and had made efforts to do so, but findings from the survey emphasized a need for change and resulted in additional outreach efforts to include the local Hispanic population in *Healthy Communities Mount Vernon*. 

Other survey results also affected future plans. Responses to questions about *integration* *and scope of the project* indicated that respondents were confused about their role in sustaining *Healthy Communities* initiatives. Many of the members believed that their commitment to the project was over at the end of the initial planning phase. In fact, as documented by meeting minutes and memos, that was how the initiative was presented when community involvement in the advisory committee was initially solicited. When the transition from planning to implementation took place, many members left the project, so new members had to be recruited to replace them. In Moses Lake, there was 50% turnover. Community leaders and DOH staff concluded that this two-part recruitment may be a necessary component of this type of intervention. If the initial recruitment had suggested that volunteers were signing up for a 5- to 7-year project, there may have been far less community involvement. In addition, the planning and implementation phases may attract people with different interests and skills, so membership turnover should be expected during the transition from planning to implementation.

## Discussion

The *Healthy Communities* project outlined in the Washington State Nutrition and Physical Activity Plan was based on the social–ecological model (5), and development of the intervention and evaluation methods were guided by understanding of obesogenic environments (5) and values of participatory research and empowerment (22-24). We find that the CHG model (14) includes most of the identified elements and may prove to be a useful resource to public health practitioners who are working with communities to facilitate policy and environmental changes.

Integral to the CHG model is the assumption that processes of community participation are crucial to effective solutions for community health problems (14). The model is rooted in the belief that sustainable solutions to many adverse health outcomes will be found only when people and organizations come together to address the social, economic, political, and environmental determinants of these outcomes. In the CHG model, the role of governmental agencies such as DOH changes from a director exerting control to a community advisor that actively participates in and drives the process of positive change. This change in role for governmental agencies represents a new paradigm for public health practitioners in community-based health promotion projects.

Similarly, the CHG model affects program evaluation. Rather than focusing only on behavior change and long-term health outcomes as measures of health promotion interventions, the CHG model provides a framework for examining the intermediate processes of the partnership as proximal outcomes. Use of the CHG model to organize information can aid in assessing processes in which individuals and organizations work together to identify and address health problems at the community level. The model can also help to guide public health workers in project management. A well-designed process evaluation guides the use of limited staff time and project funding and thus can improve efficiency and effectiveness.

The CHG model shows how the dynamic and complex interactions of community partners using community resources can lead to improved community health. The model identifies markers that can reinforce partnership activities. The markers, such as representation of community diversity and mechanisms for accountability within a collaborative process, are not ends in themselves, but the presence and strength of these markers become guides for public health practitioners in promoting community-based interventions.

The CHG model served as a guide for evaluating the *Healthy Communities* pilot projects in Washington. The model was used to develop a telephone survey tool to assess community partners' perceptions of a *Healthy Communities *project. Results from the survey provided feedback for DOH staff, university partners, and community leaders. In addition to scaled questions that addressed elements of the CHG model, several open-ended questions were important for identifying issues raised by the scaled questions. These open-ended questions provided the opportunity for respondents to voice their opinions and provided essential information for translating survey findings into meaningful directions for actions.

## Figures and Tables

**Table 1:** Events and Actions of Washington State's *Healthy Communities *Pilot Projects

**Date**	**Events and Actions**
April 2002	Moses Lake selected as pilot community for implementing Washington State Nutrition and Physical Activity Plan objectives
June–September 2002	Moses Lake advisory committee planning meetings held, community environmental inventory conducted, and action plan developed
September 2002	Moses Lake project work teams formed for trails and paths, breastfeeding, and community garden
November 2002	*Healthy Communities Moses Lake: An Action Plan to Promote Nutrition and Physical Activity* introduced to community
December 2002	First survey of Moses Lake advisory committee conducted
January 2003–present (ongoing)	Project work teams meetings held regularly and project plans implemented
November 2003	Mount Vernon selected as second pilot community for implementing state plan objectives
January–June 2004	Mount Vernon advisory committee planning meetings held, community environmental inventory conducted, and action plan developed
June 2004	Second survey of Moses Lake project teams and selected advisory committee members conducted
	*Healthy Communities Mount Vernon: An Action Plan to Promote Nutrition and Physical Activity* introduced to the community
June–September 2004	Mount Vernon project work teams formed for urban trails, school district nutrition and physical activity policy, and healthy schools pilot project
September 2004	Revised survey of Mount Vernon advisory committee conducted

**Table 2:** Sources of Data for Process Evaluation of Washington State's *Healthy Communities* Pilot Projects, 2002–2004

**Method of Data Collection**	**Source of Data**	**Data Collected**
Community inventory feedback survey	Community participants who conducted the inventory	Feedback on inventory process and findings
Meeting minutes	Advisory committee and project group meeting minutes included in quarterly progress reports	List of attendees, project updates, discussion topics, and presenters
Planning team meeting minutes for Moses Lake pilot project planning	Washington State Department of Health (DOH) staff, University of Washington, and National Park Service partners; Moses Lake leadership representative	Pilot project planning and evaluation planning discussion
Meeting evaluation surveys	Attendees at each advisory committee meeting	Logistics, format, and comments
Activity logs, interviews, staff debriefings	DOH staff and technical advisors, community leadership, and staff	Observations about project progress and partnership functions

**Table 3:** Questions in Revised Survey for *Healthy Communities* Projects That Address Elements of the Community Health Governance Model, Washington State, 2004

**Element of Model**	**No. of Survey Questions That Address Element**	**Example of Scaled Question From Mount Vernon *Healthy Communities *Survey**

**Proximal outcomes**		

Individuals are empowered	5	By participating in the Mount Vernon *Healthy Communities* project, I am making a difference in my community.
Bridge social ties: social networks increase and are strengthened	6	As a result of participating in this *Healthy Communities* project, my organization has or is planning to develop new collaborative relationships with other organizations.
Synergy: collaborations are creative and effective	5	The advisory committee worked together to identify new and creative ways to solve problems.

**Critical characteristics**		

Who: wide spectrum of community individuals and organizations are involved	2	The diversity of Mount Vernon's population is well represented by the array of people and organizations who are members of the advisory committee.
How involved: participation in all capacities is feasible	2	I feel that my responsibilities to the advisory committee were well suited to my interests and skills.
Scope of the process: ongoing planning and actions address multiple issues	5	In the planning phase, the advisory committee explored an array of issues and prioritized them based on community assessments.

**Leadership and management**		

Encourage broad and active participation	8	The advisory committee is effective at providing orientation to new partners as they join the committee.
Ensure influence and control are broadly based	4	I have been included in the decision-making process of the advisory committee.
Facilitate group processes	4	In advisory committee discussions, members used language that was common to everyone and easy to understand.
Scope of process expands incrementally, remains integrated	4	As a group, we are building skills and expertise to carry out the objectives and meet the goals of the Mount Vernon *Healthy Communities* project.

**Table 4:** Resources for Design of Survey Tool to Evaluate Community Partnerships for Health Promotion Projects

**Resource**	**Comments**
Social capital index (15)	Model for measuring social capital based on indicators of trust, involvement, and reciprocity
Evaluating partnerships (7)	Outline of criteria applied to index partnership management and perceived costs and benefits
Measuring perceptions of multiple levels of control (16)	Statements applied to index individual and community levels of empowerment
Assessing principles of partnership (17)	Survey questions based on 10 principles of community–campus partnership
Partnership synergy self-assessment tool (18)	Online tool based on elements outlined in community healthy governance model (14,19)
Community partnership stakeholders questionnaire (20)	Survey questions addressing stakeholder view of participation and outcomes based on a study of a community partnership for healthy personnel education
Community coalition action theory (21)	Model includes elements of coalition membership and processes that create synergy for community capacity and change outcomes
